# Prognostic nomogram for cancer-specific survival in patients with intrahepatic cholangiocarcinoma after hepatectomy: A population study of 919 patients

**DOI:** 10.3389/fsurg.2022.1025521

**Published:** 2023-01-06

**Authors:** Gaobo Huang, Weilun Song, Yanchao Zhang, Bingyi Ren, Yi Lv, Kang Liu

**Affiliations:** ^1^ National Local Joint Engineering Research Center for Precision Surgery and Regenerative Medicine, First Affiliated Hospital of Xi'an Jiaotong University, Xi'an, China; ^2^ Department of Oncology, Xi'an No.3 Hospital, Xi'an, China; ^3^ Department of Hepatobiliary Surgery, First Affiliated Hospital of Xi'an Jiaotong University, Xi'an, China

**Keywords:** nomogram, cancer-specific survival, intrahepatic cholangiocarcinoma, hepatectomy, prognosis

## Abstract

**Background and Aims:**

Intrahepatic cholangiocarcinoma has an increasing global incidence and mortality rate. Hepatectomy is still the most effective curative treatment for patients with ICC, but the prognosis of patients with ICC is still poor even after curative resection. This study aimed to incorporate important factors obtained from SEER database to construct and validate a nomogram for predicting the cancer-specific survival of patients with ICC after hepatectomy.

**Methods:**

We obtained patient data from SEER database. The nomogram was constructed base on six prognostic factors for predicting CSS rates in ICC patients. The nomogram was validated by C-index, ROC curve and calibration curves.

**Results:**

A total of 919 patients with ICC after hepatectomy between 2000 and 2018 were included in this study. A nomogram based on six independent prognostic factors (Black race, AJCC T, AJCC N, AJCC M, chemotherapy and PLNR ≥ 0.15) was developed for the prediction of CSS at 3 and 5 years. The C-index of the nomogram and AJCC stage system were 0.709 and 0.657 in the training cohort respectively. The 3- and 5-year AUCs of nomogram were 0.744 and 0.75 in the training cohort. The calibration plots indicated that there was good agreement between the actual observations and predictions.

**Conclusions:**

In conclusion, we constructed and validated a nomogram for predicting the 3- and 5-year CSS in ICC patients after hepatectomy. We have confirmed the precise calibration and acceptable discrimination power of our nomogram. The predictive power of this nomogram may be improved by considering other potential important factors and also by external validation.

## Introduction

Intrahepatic cholangiocarcinoma (ICC), second only to hepatocellular carcinoma (HCC) as the most common liver cancer, has an increasing global incidence and mortality rate (1–3). ICC arises from the epithelial layer of the second-degree biliary tract, and has a high degree of malignancy (4, 5). Hepatectomy is still the most effective curative treatment for patients with ICC (6). Unfortunately, even after curative resection, the prognosis of patients with ICC remains poor with a five-year overall survival rate of only 20%–35% (7). Therefore, it is necessary to integrate multiple prognostic factors into an easy-to-use predictive system to better inform surgeons and patients with ICC.

Presently, the most commonly used classification system for patients with ICC is the American Joint Committee on Cancer (AJCC) TNM staging system (8). However, the AJCC system is not easy to apply and it neglects many significant prognostic factors such as race, age, and grade (9). A nomogram is a prognostic predictive tool that creates a user-friendly graph based on a statistical model (10). It can be used to calculate the probability of a clinical outcome by considering the prognostic weight of each factor. This tool is been widely used in clinical decision-making (11–13).

This study aimed to incorporate important factors obtained from the Surveillance, Epidemiology, and End Results (SEER) database to construct and validate a nomogram for predicting cancer-specific survival (CSS) in patients with ICC after hepatectomy. To the best of our knowledge, the nomogram of this study contains the most patients with ICC (919 patients) who have undergone hepatectomy. We compared our model with the AJCC staging system to determine whether it provides a more accurate prediction.

## Materials and methods

### Ethics statement

Our data were obtained from the SEER database with a signed data agreement (11187-Nov2021). The approval and informed consent of the institutional review committee were exempted since the SEER database is a public database and provides open access for anyone who has registered an account and signed the authorization.

### Study population

We obtained patient data from the SEER Research Plus Data, 18 Registries, Nov 2020 Sub (2000–2018) incidence database using SEER*Stat version 8.3.9. The data were obtained from the International Classification of Diseases for Oncology 3rd edition (ICD-O-3), primary site code C22.1 (intrahepatic bile duct), and histological/behavior code 8160.3 (cholangiocarcinoma). The exclusion criteria were as follows: (1) no hepatectomy performed; (2) incomplete basic information (age, race, and sex); (3) incomplete tumor information (AJCC T, N, M, grade, and lymph nodes examined). The staging system used was the 8th edition of the AJCC system which was calculated from the 6th or 7th edition TNM stages and included other characteristics such as tumor size.

**Figure 1:**
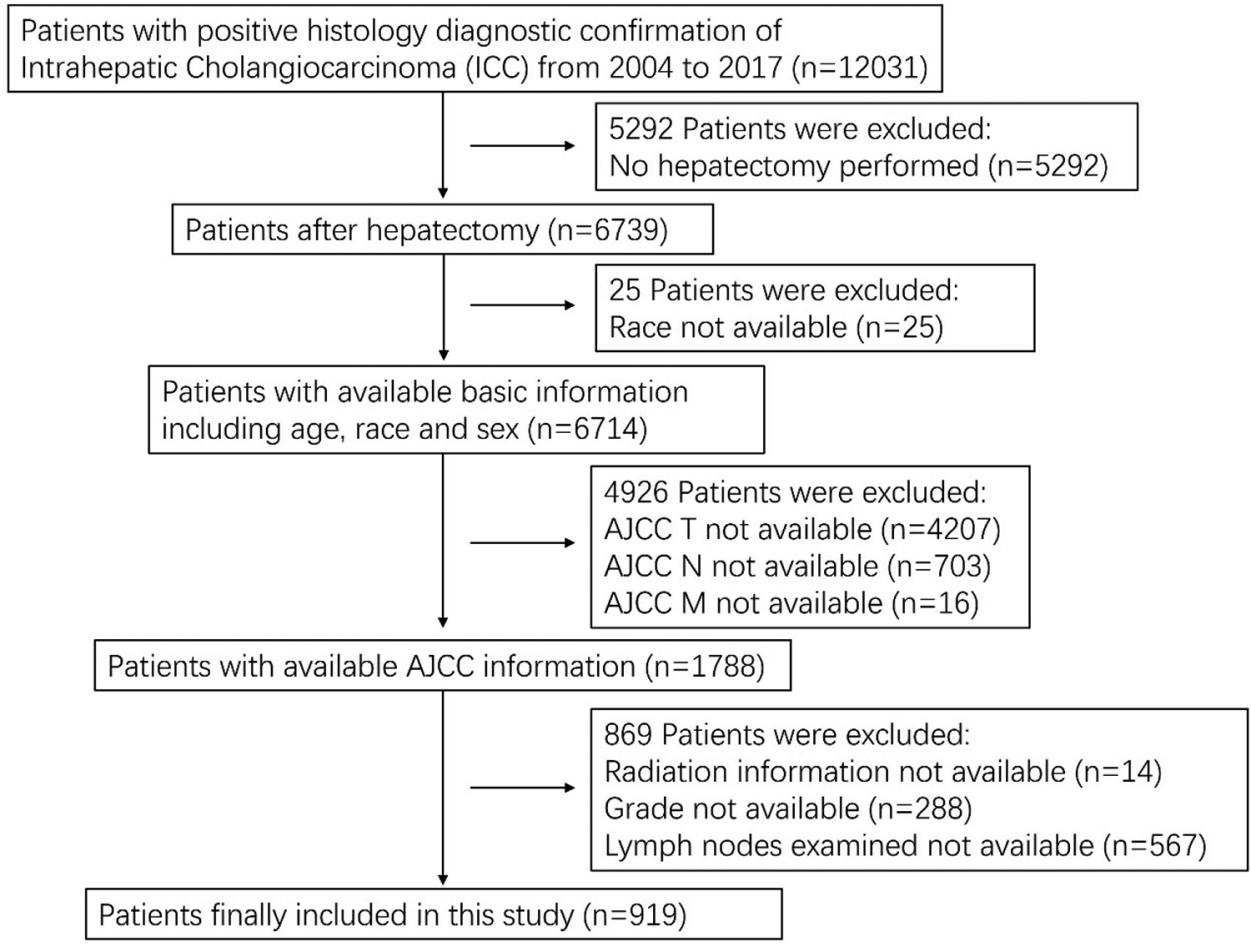
Flow chart of patients with ICC included in this study.

A total of 12,031 patients with ICC were identified and 919 patients who underwent hepatectomy were included in this study. A flow chart for selecting the research samples is shown in Figure 1. The following additional data (variables) were used in the analysis: patient sex, age and race; AJCC staging for the extent of tumor (T), the extent of spread to lymph nodes (N), presence of metastasis (M), tumor grade, radiation (Y/N), chemotherapy (Y/N), number of examined regional nodes, number of positive regional nodes, months of survival, and vital status records. Positive lymph node ratio (PLNR) was calculated by dividing the number of lymph nodes examined by the number of positive lymph nodes.

### Statistical analysis

The PLNR cutoff point was analyzed using an X-tile plot (14). A two-population model was implemented to segregate the patients into two groups according to PLNR (PLNR < 0.15 and PLNR ≥ 0.15).

For nomogram construction and validation, we randomly divided all patients with ICC after hepatectomy into training (*n* = 643) and validation (*n* = 276) cohorts at a ratio of 7:3 (15, 16). Multivariate cox proportional hazards regression analysis was performed to identify variables (*P* < 0.05) that significantly affected CSS in the training group. Using these identified prognostic factors, we constructed a nomogram for predicting the three- and five-year CSS rates in patients with ICC after hepatectomy.

The nomogram was internally validated in the training cohort and externally validated in the validation cohort. To evaluate the discriminative ability of the nomogram, we used the concordance index (C-index) and receiver operating characteristic (ROC) curve to assess the area under the curve (AUC) (17, 18). A C-index or AUC of 0.5 indicates a discrimination ability that is no better than chance, whereas an AUC of 1.0 indicates a perfect discrimination ability (19). Calibration curves were constructed using a bootstrap approach, with 500 resamples, to compare the predicted CSS with the CSS observed in the study.

All statistical analyses were performed using SPSS (version 24.0; SPSS, Chicago, IL, USA) and R software (version 4.1.2; http://www.r-project.org/). A *P* value of < 0.05 was considered to indicate statistical significance.

## Results

### Patient characteristics

A total of 919 patients with ICC who underwent hepatectomy between the years 2000 and 2018 were included in this study. The training and validation cohorts consisted of 643 and 276 cases, respectively, which were selected using the random split-sample method (split ratio: 7:3). In the total cohort of patients with ICC after hepatectomy, the majority of patients were female (52.8%), under 65 years of age (59.4%) and white (66.5%). Furthermore, most of the patients had T1 (35.8%), N0 (66.6%), and M0 (92.5%), Patients with grade I and II tumor differentiation degree accounted for 67.7% of all cases. A large proportion of the patients did not receive radiation therapy (82.6%) but received chemotherapy (51.0%). Most patients with ICC after hepatectomy had a PLNR of < 0.15. The characteristics of patients with ICC after hepatectomy in the training and validation cohorts were similar to those in the total cohort (Table 1).

**Table 1:** ICC patient characteristics in the study.

Characteristics	Total cohort	Training cohort	Validation cohort
	919 (100%)	643 (70%)	276 (30%)
**Sex**			
Male	434 (47.2%)	306 (47.6%)	128 (46.4%)
Female	485 (52.8%)	337 (52.4%)	148 (53.6%)
**Age**			
<65	487 (53.0%)	343 (53.3%)	144 (52.2%)
≥65	432 (47.0%)	300 (46.7%)	132 (47.8%)
**Race**			
W	733 (79.8%)	515 (80.1%)	218 (79.0%)
B	66 (7.18%)	41 (6.38%)	25 (9.06%)
AI	9 (0.98%)	5 (0.78%)	4 (1.45%)
API	111 (12.1%)	82 (12.8%)	29 (10.5%)
**AJCC T**			
T1	329 (35.8%)	223 (34.7%)	106 (38.4%)
T2	270 (29.4%)	187 (29.1%)	83 (30.1%)
T3	184 (20.0%)	127 (19.8%)	57 (20.7%)
T4	136 (14.8%)	106 (16.5%)	30 (10.9%)
**AJCC N**			
N0	612 (66.6%)	426 (66.3%)	186 (67.4%)
N1	307 (33.4%)	217 (33.7%)	90 (32.6%)
**AJCC M**			
M0	850 (92.5%)	596 (92.7%)	254 (92.0%)
M1	69 (7.51%)	47 (7.31%)	22 (7.97%)
**Grade**			
I + II	622 (67.7%)	430 (66.9%)	192 (69.6%)
III + IV	297 (32.3%)	213 (33.1%)	84 (30.4%)
**Radiation**			
No	759 (82.6%)	532 (82.7%)	227 (82.2%)
Yes	160 (17.4%)	111 (17.3%)	49 (17.8%)
**Chemotherapy**			
No	450 (49.0%)	304 (47.3%)	146 (52.9%)
Yes	469 (51.0%)	339 (52.7%)	130 (47.1%)
**PLNR**			
<0.15	650 (70.7%)	450 (70.0%)	200 (72.5%)
≥0.15	269 (29.3%)	193 (30.0%)	76 (27.5%)

W, White; B, Black; AI, American Indian/Alaska native; API, Asian or Pacific Islander; PLNR, positive lymph node ratio.

### Screening for prognostic factors of CSS

**Figure 2:**
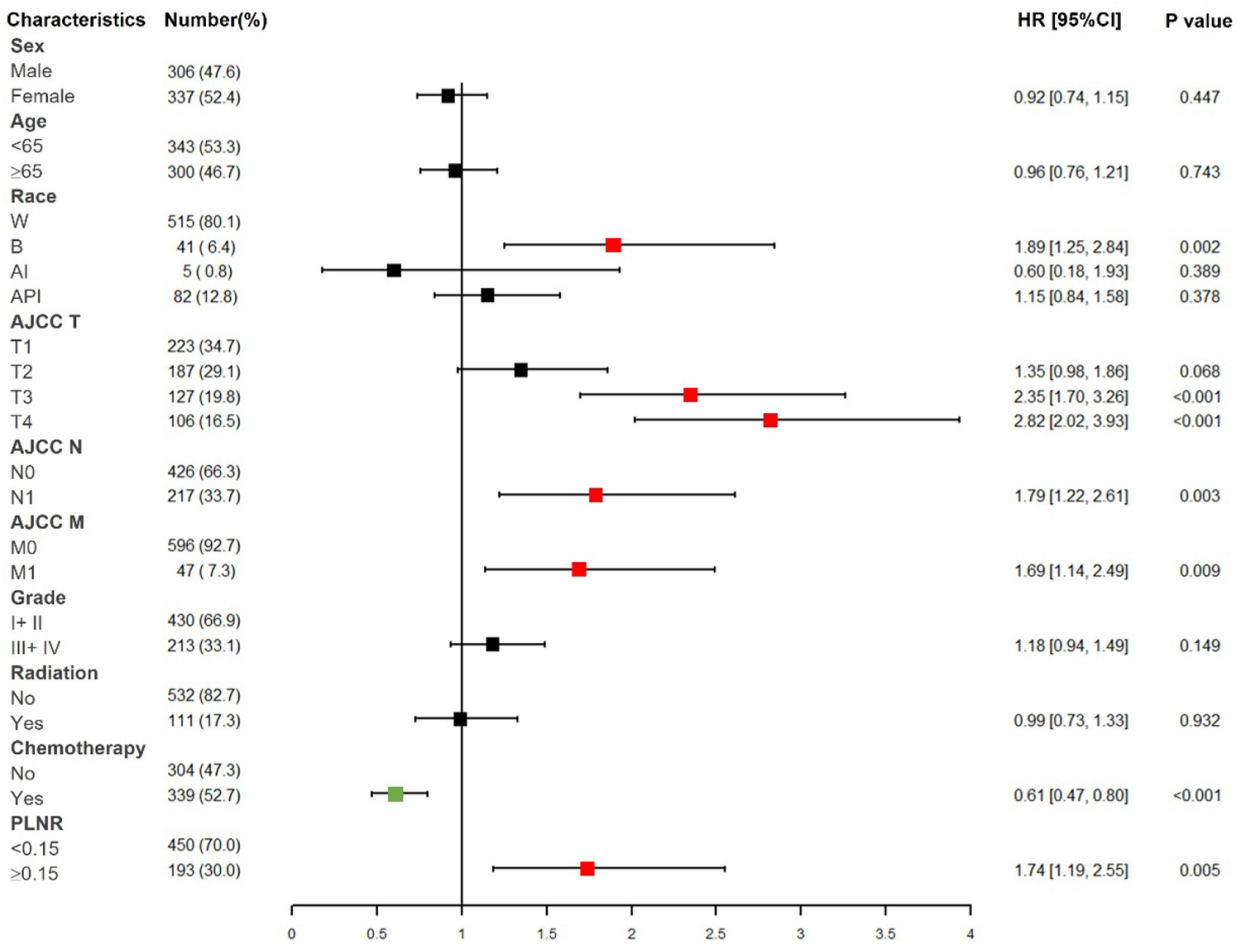
Forest plot of multivariate cox regression analysis for CSS in patients with ICC after hepatectomy. W, White; B, Black; AI, American Indian/Alaska native; API, Asian or Pacific Islander; PLNR, positive lymph node ratio.

We identified six independent prognostic factors in the training cohort based on univariate and multivariate cox proportional hazard regression analyses. Black race [hazard ratio (HR) = 1.885, *P* < 0.01], AJCC T3/T4 (HR = 2.352/2.819, *P* < 0.001), AJCC N1 (HR = 1.787, *P* < 0.01), AJCC M1 (HR = 1.685, *P* < 0.01), chemotherapy (HR = 0.612, *P* < 0.001) and PLNR ≥ 0.15 (HR = 1.738, *P* < 0.01) were significantly associated with CSS in patients with ICC after hepatectomy (Figure 2 and Table 2).

**Table 2:** Univariate and multivariate cox regression analysis based on all variables for ICC patient cancer-specific survival (training cohort).

Characteristics	Univariate analysis		Multivariate analysis	
	HR (95% CI)	*P* value	HR (95% CI)	*P* value
**Sex**				
Male	Reference		Reference	
Female	0.842 (0.680–1.043)	0.116	0.917 (0.735–1.145)	0.446
**Age**				
<65	Reference		Reference	
≥65	0.916 (0.738–1.138)	0.432	0.962 (0.763–1.213)	0.743
**Race**				
W	Reference		Reference	
B	1.546 (1.040–2.299)	0.031*	1.885 (1.249–2.843)	**<0**.01**
AI	0.812 (0.260–2.539)	0.721	0.596 (0.184–1.932)	0.389
API	1.157 (0.847–1.579)	0.357	1.151 (0.841–1.576)	0.378
**AJCC T**				
T1	Reference	** **	Reference	** **
T2	1.614 (1.193–2.183)	**<0**.01**	1.349 (0.978–1.859)	0.067
T3	2.556 (1.887–3.461)	**<0**.001***	2.352 (1.697–3.259)	**<0**.001***
T4	3.544 (2.605–4.821)	**<0**.001***	2.819 (2.022–3.930)	**<0**.001***
**AJCC N**				
N0	Reference		Reference	
N1	2.823 (2.265–3.518)	**<0**.001***	1.787 (1.223–2.612)	**<0**.01**
**AJCC M**				
M0	Reference		Reference	
M1	2.295 (1.621–3.249)	**<0**.001***	1.685 (1.141–2.488)	**<0**.01**
**Grade**				
I + II	Reference		Reference	
III + IV	1.281 (1.023–1.603)	**0**.031*	1.184 (0.941–1.489)	0.149
**Radiation**				
No	Reference		Reference	
Yes	1.005 (0.769–1.314)	0.969	0.987 (0.734–1.326)	0.932
**Chemotherapy**				
No	Reference		Reference	
Yes	1.056 (0.851–1.310)	0.617	0.612 (0.470–0.796)	**<0**.001***
**PLNR**				
<0.15	Reference		Reference	
≥0.15	3.001 (2.401–3.747)	**<0**.001***	1.738 (1.186–2.547)	**<0**.01**

W, White; B, Black; AI, American Indian/Alaska native; API, Asian or Pacific Islander; PLNR, positive lymph node ratio. *P < 0.05; **P < 0.01; ***P < 0.001.

### Nomogram construction

**Figure 3:**
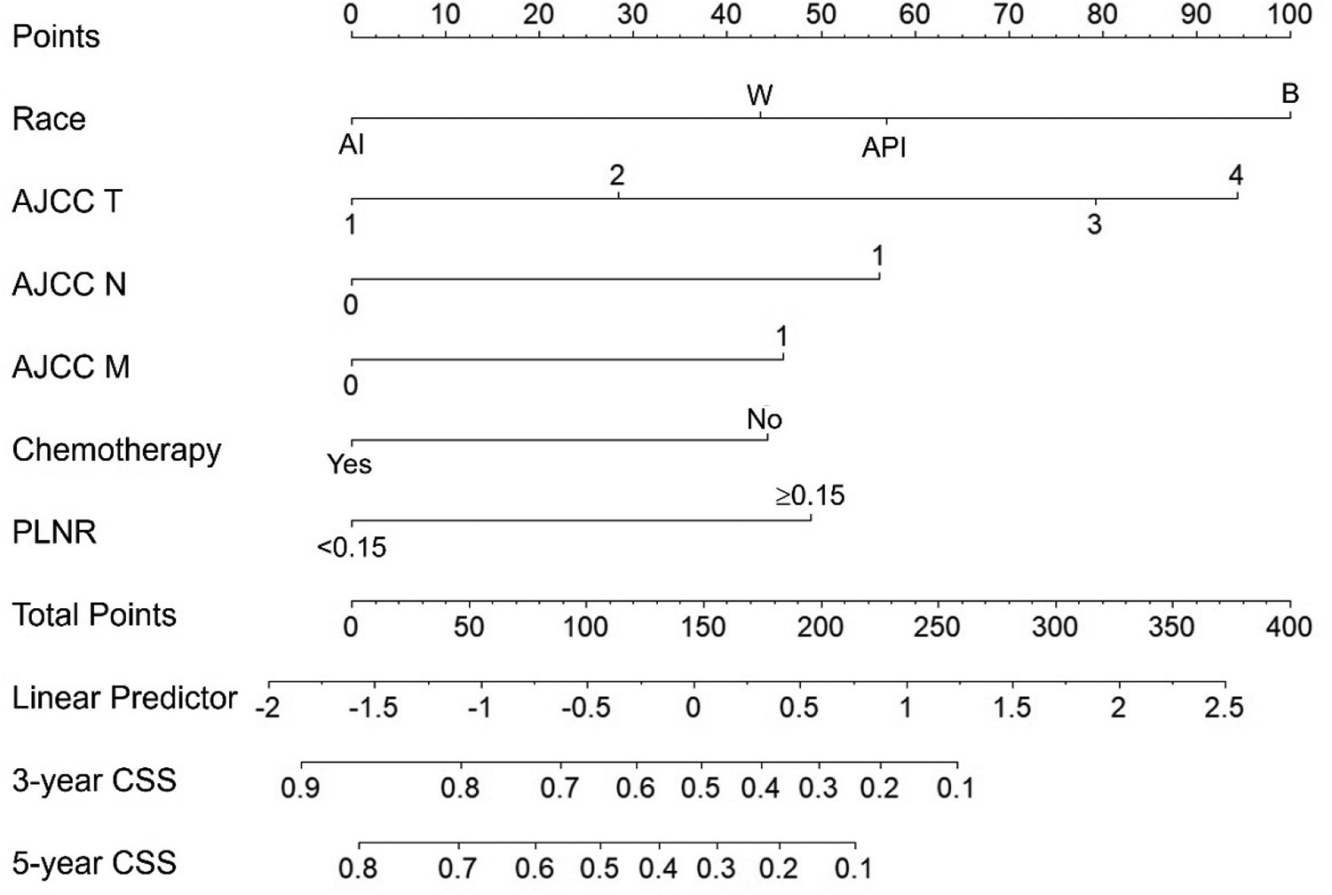
The nomogram predicting 3- and 5- years CSS in patients with ICC after hepatectomy.

As shown in Figure 3, our nomogram was constructed based on independent prognostic factors in the training cohort to predict 3- and 5- years CSS of patients with ICC after hepatectomy. The nomogram demonstrated that patient race contributed the most to the prognosis, followed by AJCC T, AJCC N, PLNR, AJCC M and chemotherapy. When using the nomogram, every patient with ICC obtained scores based on the level of each independent prognostic factor. The total score was calculated by adding the scores for each factor. The 3- and 5- years CSS could be estimated by the total points.

### Nomogram validation

**Figure 4:**
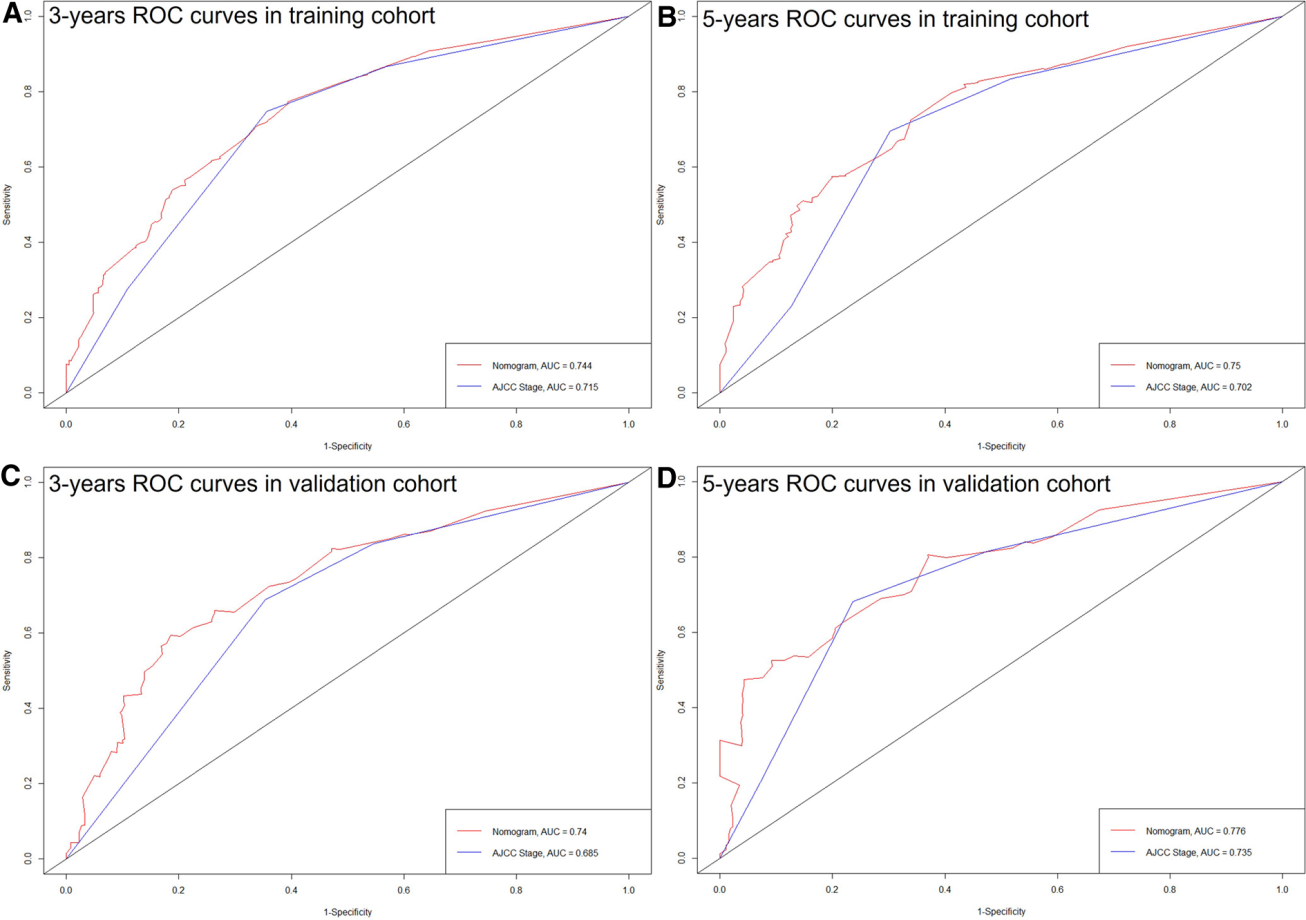
ROC curves of the nomogram and the AJCC staging system. (**A**) 3 years in training cohort; (**B**) 5 years in training cohort; (**C**) 3 years in validation cohort; (**D**) 5 years in validation cohort.

**Figure 5:**
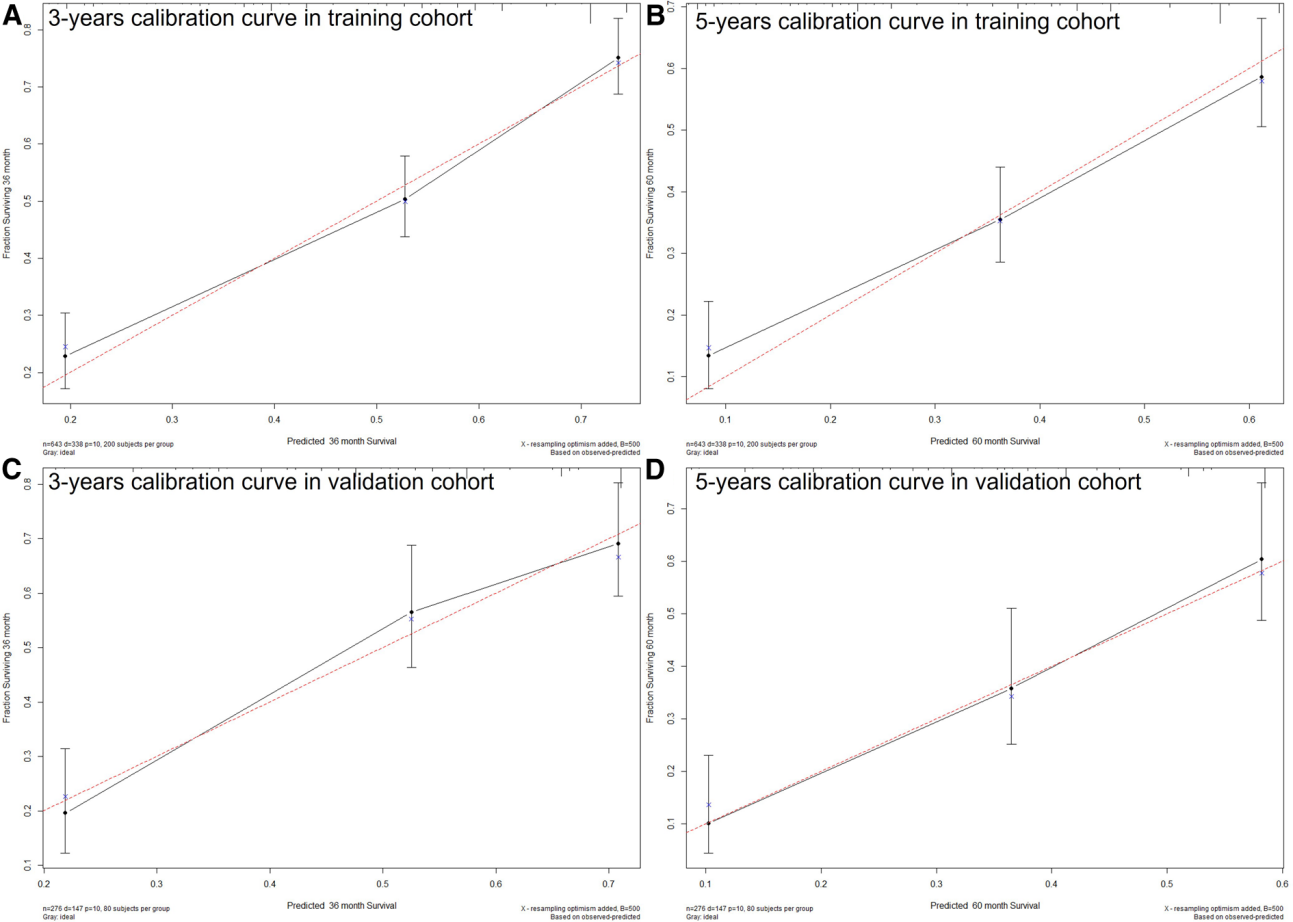
The calibration plots show excellent agreement between observed outcomes and predicted survival probabilities. (**A**) 3 years in training cohort; (**B**) 5 years in training cohort; (**C**) 3 years in validation cohort; (**D**) 5 years in validation cohort.

The C-index of the nomogram in this study (training cohort = 0.709, validation cohort = 0.683) was higher than that of the AJCC staging system (training cohort = 0.657, validation cohort = 0.657). And the AUCs of the nomogram were also higher than the AJCC staging system in both training (3-year AUC: 0.744 vs. 0.715, 5-year AUC: 0.75 vs.0.702, Figures 4A,B) and validation (3-year AUC: 0.74 vs. 0.685, 5-year AUC: 0.776 vs. 0.735, Figures 4,D) cohorts. The C-index and AUCs demonstrated the acceptable discrimination performance of our nomogram. Furthermore, the calibration plots for the CSS at 3- and 5-years showed a good agreement between prediction by nomogram and actual observation (Figure 5).

## Discussion

Although the AJCC staging system is widely used for predicting prognosis in patients with ICC, it has inherent defects because it neglects many additional risk factors other than the TNM factors. Moreover, the AJCC staging system has not yet been specifically developed for postoperative prognostic prediction (8). It is widely known that a model has relatively good discrimination if its C-index and AUC exceed 0.7 (20). Consequently, we observed that the C-index of the AJCC staging system was 0.657 for survival prediction in both the training and validation cohorts.

Nomograms have been shown to be more accurate and user-friendly than the conventional staging system in many cancers (21, 22), and liver resection is the only established curative method for ICC patients. Therefore, we constructed a more comprehensive model based on a combination of various risk factors to better predict the prognosis of patients with ICC after hepatectomy. The nomogram in this study performed well in predicting cancer-specific survival, and its prediction was supported by the C-index (0.744) and calibration curve.

Other researchers have made efforts in the past. In 2013, Wang et al. established a prognosis nomogram for ICC after partial hepatectomy and focused on the influence of laboratory examination results such as AFP, CEA, and CA-199 in patients with ICC after hepatectomy (8). It was a single-center study of 367 patients. Our study has the advantage of being a population based study with a larger sample size (919 patients). Similarly, Yuan et al*.* developed a prognostic nomogram for patients with ICC in 2021 (23). The biggest difference was that they included all patients with ICC no matter whether those patients underwent hepatectomy or not. As we know, hepatectomy is still the most effective curative treatment for patients with ICC. Therefore, the patients with ICC who did not undergo surgery were expected to have worse survival outcomes. Furthermore, our study found that race was an important prognostic factor, which was not included in the Yuan et al. study.

The effects of racial factors on patients with ICC have rarely been studied. Firas et al. (24) found that African Americans had a lower incidence of ICC than Whites. Linlin et al. (25) reported that race did not influence the prognosis of patients with ICC. In our study, black patients had a worse prognosis than white (HR = 1.885, *P* < 0.01). However, the reason for this is unclear. Lee et al. (26) reported that black patients with cholangiocarcinoma had a lower surgical rate than white patients did (odd's ratio: 0.73; *P* < 0.001). This could be due to socioeconomic reasons, such as uninsured status and Medicaid insurance. However, there were no racial or socioeconomic differences in the multimodal therapy once the patients accessed surgical care. In our study, all patients underwent surgery, although the number of black patients after surgery was few (66/919). Therefore, this result needs to be further verified in future studies. The possible influence of genetics behind this disparity is also worth exploring.

Furthermore, the benefits of chemotherapy for surgically resected ICC remain poorly defined. A randomized study indicated that chemotherapy improved the survival of patients with ICC (6.5 months vs. 2.5 months) but it was not significant (*P* = 0.1) (27). John et al. (28) proposed that chemotherapy for resected ICC should be strongly considered for tumors harboring high-risk features, their conclusion was based on the National Cancer Database. Primrose et al. (29) discovered that capecitabine can improve overall survival in patients with resected biliary tract cancer when used as adjuvant chemotherapy following surgery and could be considered a standard of care. In our study, chemotherapy significantly improved the prognosis of patients with ICC who have undergone resection (HR = 0.612, *P* < 0.001), which verified the benefit of chemotherapy in patients with ICC after hepatectomy.

It is well known that positive lymph node (PLN) is an important negative prognostic indicator for cancer patients. However, owing to the different numbers of examined lymph nodes, the number of PLN could vary among patients with a similar prognosis (30, 31). Therefore, we chose PLNR as the research object in our study, which combined the number of PLN and the number of examined lymph nodes. We identified that PLNR ≥ 0.15 is a significant poor prognostic factor in patients with ICC who have undergone resection (HR = 1.738, *P* < 0.01).

Several nomograms have been constructed to predict the prognosis of patients with ICC after hepatectomy (8, 32, 33), but our study had a larger sample size and the data were population based. Our study has some limitations. First, this large-sample study was based on the SEER database, which may have inherent bias. Second, our study included 6,739 patients with ICC after hepatectomy initially. However, only 919 patients were finally included after rigor screening, which may weaken the external validity of our nomogram. Third, our nomogram was internally validated. It would be better to validate it externally using other populations.

## Conclusion

In this study, we constructed and validated a nomogram for predicting the three- and five-year CSS in patients with ICC after hepatectomy. Six independent prognostic factors were identified, which are as follows: Black race, AJCC T, AJCC N, AJCC M, chemotherapy, and PLNR ≥ 0.15. The precise calibration and acceptable discrimination power of the nomogram were verified. The nomogram could be improved by further external validation and including additional potential factors that were not available in the SEER database.

## Data Availability

The original contributions presented in the study are included in the article/Supplementary Material, further inquiries can be directed to the corresponding author/s.
